# Chemotherapy-free innovations in locally advanced head and neck cancer: a comprehensive review

**DOI:** 10.3389/fonc.2025.1552337

**Published:** 2025-04-22

**Authors:** Shatha Abu Taha, Taher Abu Hejleh, Mostafa ElHaddad, Akram Al-Ibraheem, Ahmed Abbasi, Abdulrahman Sumaida, Ahmad Bushehri, Ahmad Mostafa, Bassem Youssef, Ibrahim Alotain, Ibrahim Abu-Gheida, Mohammed Aldehaim, Majed Alghamdi, Mohamed Shelan, Mohammed Al Dohan, Maysa Al-Hussaini, Nadeem Pervez, Shoukri Temraz, Saad Alrashidi, Wael El-Sheshtawy, Zahid Al-Mandhari, Hamza Ghatasheh, Ali Hosni, Issa Mohamad

**Affiliations:** ^1^ Department of Radiation Oncology, King Hussein Cancer Center, Amman, Jordan; ^2^ Department of Medical Oncology, King Hussein Cancer Center, Amman, Jordan; ^3^ Clinical Oncology Department, Kasr Al-Ainy Center of Clinical Oncology and Nuclear Medicine, Kasr Al-Ainy School of Medicine, Cairo University, Cairo, Egypt; ^4^ Department of Nuclear Medicine, King Hussein Cancer Center, Amman, Jordan; ^5^ Department of Radiation Oncology, Aga Khan University Hospital, Karachi, Pakistan; ^6^ Oncology: Radiation Oncology, Tawam Hospital, Al Ain, United Arab Emirates; ^7^ Department of Radiation Oncology, Kuwait Cancer Control Center, Kuwait, Kuwait; ^8^ Clinical oncology department, Minia Oncology, Center, Minia, Egypt; ^9^ Department of Radiation Oncology, American University of Beirut Medical Centre, Beirut, Lebanon; ^10^ Department of Radiation Oncology, King Fahad Specialist, Dammam, Saudi Arabia; ^11^ Department of Radiation Oncology, Burjeel Medical City, Abu Dhabi, United Arab Emirates; ^12^ Department of Radiation Oncology, King Faisal Specialist Hospital and Research Center, Riyadh, Saudi Arabia; ^13^ College of Medicine, Alfaisal University, Riyadh, Saudi Arabia; ^14^ Radiation Oncology, Princess Noorah Oncology Center, King Abdulaziz Medical City, Ministry of National Guard Health Affairs-Western Region, Jeddah, Saudi Arabia; ^15^ Collage of Medicine, King Saud Bin Abdulaziz University for Health Science, Jeddah, Saudi Arabia; ^16^ Department of Radiation Oncology, Inselspital Bern, University of Bern, Bern, Switzerland; ^17^ Department of Radiation Oncology, King Fahad Medical City, Riyadh, Saudi Arabia; ^18^ Department of Cell Therapy and Applied Genomics, King Hussein Cancer Center, Amman, Jordan; ^19^ Department of Radiation Oncology, United Arab Emirates (UAE) University, Al Ain, United Arab Emirates; ^20^ Clinical Oncology Department, Mansoura University hospital, Mansour, Egypt; ^21^ Department of Radiation Oncology, Comprehensive Cancer Center, King Fahad Medical City, Riyadh, Saudi Arabia; ^22^ Department of Clinical Oncology, Al-Azhar University, Cairo, Egypt; ^23^ Department of Radiation Oncology, Sultan Qaboos Comprehensive Cancer Care and Research Centre, Muscat, Oman; ^24^ Radiation Medicine Program, Princess Margaret Cancer Centre, University Health Network, University of Toronto, Toronto, ON, Canada

**Keywords:** head and neck cancer, concurrent chemoradiation, altered fractionation, immunotherapy, targeted therapy, hyperthermia, nanoparticle

## Abstract

The treatment of locally advanced head and neck squamous cell carcinoma (LA-HNSCC) has traditionally relied on a multimodal approach, combining surgery, radiation therapy (RT), and chemotherapy. While chemotherapy plays a critical role in improving cure rates and functional outcomes, its substantial toxicity remains a major concern, particularly in older patients. These challenges are especially relevant for those who are unfit for chemotherapy or decline conventional concurrent chemoradiotherapy (CCRT), highlighting the need for alternative therapeutic options. Many patients are at high risk for severe side effects, often preventing them from completing the full chemotherapy regimen. This review explores alternative strategies to definitive CCRT of carcinomas of the larynx, hypopharynx and oropharynx, aiming to optimize treatment outcomes while minimizing toxicity. We discuss altered fractionation strategies as a promising alternative to conventional RT, offering a balance between treatment efficacy and quality of life. Additionally, we examine emerging approaches, including the combining of targeted therapies, immunotherapy, hyperthermia, photodynamic therapy and nanoparticle-based treatments with RT, which provide alternative or complementary options to traditional therapies in the management of LA-HNSCC.

## Introduction

Locally advanced head and neck squamous cell carcinoma (LA-HNSCC) is a major global health challenge, accounting for 7.6% of all cancers and 4.8% of cancer-related deaths worldwide (1, 2). Historically linked to tobacco and alcohol consumption, the increasing prevalence of human papillomavirus (HPV)-associated oropharyngeal cancers has contributed to a demographic shift, with a rising incidence observed among younger populations (3). Projections indicate a 30% annual increase in HNSCC cases by 2030, highlighting the urgent need for effective treatment options (4).

The management of non-metastatic LA-HNSCC has traditionally involved a multidisciplinary approach, combing surgery, radiation therapy (RT), and systemic treatments such as chemotherapy or targeted therapies (5). In several HNSCC subsites, definitive concurrent chemoradiation (CCRT) therapy yields survival outcomes comparable to curative-intent surgery, often preserving superior functional outcomes, particularly in laryngeal cancer (6). Cisplatin remains the standard of care when combined with RT. Despite its effectiveness, cisplatin is associated with significant toxicities, including nephrotoxicity, ototoxicity and neurotoxicity (7), which pose substantial challenges, particularly for older patients (8, 9). Notably, one-third of LA-HNSCC cases occur in patients over 70, who face higher risks of serious complications due to pre-existing comorbid conditions (10–12). In response to these challenges, there is growing interest in alternative strategies that optimize treatment efficacy while minimizing toxicity. Alternative approaches such as altered RT regimens (e.g. hyperfractionation (HFX)) and combining the targeted therapies (e.g. cetuximab), immunotherapy (e.g. pembrolizumab), hyperthermia, photodynamic therapy or nanoparticle-based treatments with RT, offer promising alternatives interventions to conventional CCRT which are strategies of particular interest in patients who are unfit for systemic chemotherapy.

This review focuses on potential chemotherapy-free approaches for managing LA-HNSCC, especially tumors of the larynx, hypopharynx, and oropharynx. We highlight the role of altered RT regimens and the potential of novel therapies like targeted agents, immunotherapy, hyperthermia, and nanoparticle-based treatments. We aim to highlight their clinical indications, integration into multidisciplinary care, and potential to improve patient outcomes. While these strategies show promise, further clinical validation is needed to establish their role as standard practice in the management of LA-HNSCC.

## Evidence acquisition

A comprehensive literature search was conducted using PubMed, EMBASE, and MEDLINE to identify relevant studies on chemotherapy-free innovations in the treatment of LA-HNSCC. The search was performed utilizing a combination of Medical Subject Headings terms and keywords such as “head and neck cancer,” “immunotherapy,” “radiotherapy,” and “targeted therapy”. Additional filters were applied to focus on clinical trials, systematic reviews, and high-impact studies published in peer-reviewed journals. Reference lists of key articles were also screened to identify additional relevant studies. Only English-language articles were included in this review.

## The role of chemotherapy in LA-HNSCC

Chemotherapy plays a key role in the multimodal treatment of LA-HNSCC, improving both cure rates and functional outcomes. It is typically used in combination with RT either as CCRT or induction therapy before definitive CCRT. In cases of unresectable LA-HNSCC, CCRT can provide the option of organ preservation without compromising the curative intent of treatment (13).

Concurrent chemotherapy enhances the efficacy of RT by acting as a radiosensitizer (14, 15). The MACH-NC meta-analysis of 101 randomized controlled trials (RCTs) involving 18,951 patients with non-metastatic HNSCC (mainly stage III-IV) assessed chemotherapy’s impact on survival. The analysis found that CCRT provided a 6.5% absolute improvement in 5-year OS (HR=0.83, 95% CI: 0.79–0.86; p < 0.0001) compared to chemotherapy given as induction or adjuvant treatment rather than concurrently with RT. Induction chemotherapy showed a 2.2% benefit (HR=0.96, 95% CI: 0.90–1.01; p=0.14), while adjuvant chemotherapy showed no benefit (HR = 1.02 [0.92; 1.13]). These findings confirm CCRT as the most effective strategy for reducing cancer-related mortality. Platinum-based mono-chemotherapy was more effective than other single-agents, and no OS difference was found between mono and combination chemotherapy (16).

High-dose cisplatin (100 mg/m² on weeks 1, 4, and 7) remains the preferred concurrent systemic therapy, improving OS and DFS (17). Alternatives include carboplatin/5-fluorouracil, which improve PFS (18) and OS (19), and low-dose weekly cisplatin (40 mg/m²), which offers acceptable toxicity and survival outcomes (20, 21). The ongoing NRG-HN009 trial (ClinicalTrials.gov identifier: NCT05050162) is a phase II/III study comparing the efficacy and toxicity of two cisplatin-based CCRT regimens for LA-HNSCC: low-dose weekly cisplatin (40 mg/m²) with standard high-dose cisplatin (100 mg/m² every three weeks). This trial aims to assess toxicity and determine if reducing the dose of cisplatin does not compromise OS outcomes.

## The challenges of chemotherapy

The significant toxicities of chemotherapy often limit its use, especially in patients with LA-HNSCC. Cisplatin is known to be associated with nephrotoxicity, with studies reporting grade 2-3 nephrotoxicity in 33% of patients, with 17% of patients unable to complete the planned regimen due to these complications (22). Additionally, it causes ototoxicity, leading to hearing loss and tinnitus (23), with an incidence of 36% in adult cancer patients (24). Neurotoxicity, particularly chronic sensory neuropathy, is another dose dependent side effect of cisplatin (25). Of note, effective prevention or treatment for these toxicities remains unsatisfactory (26).

Carboplatin, a commonly used alternative to cisplatin, also has its own toxicity profile, with myelosuppression being a frequent side effect. A study on concurrent carboplatin and RT showed a risk of thrombocytopenia and neutropenia in 34% and 28%, respectively (27).

The combination of chemotherapy and RT is also associated with challenges linked to treatment adherence. One study found 84% of patients who were non-adherent to RT were also receiving concurrent chemotherapy, highlighting the challenges of the combined approach (28). For weekly cisplatin, 30-60% of patients miss at least one cycle (29), while for high-dose cisplatin given every three weeks, adherence to all planned cycles ranges from 61-85% (30, 31).

The use of chemotherapy in older (>70 years) population, is associated with longer hospital stays, readmissions, and chemotherapy-induced toxicities (32, 33). Additionally, chemotherapy in older adults increases the risk of severe toxicities, including grade ≥3 pharyngeal/laryngeal toxicity, feeding tube dependency, and treatment-related mortality (34). The MACH-NC meta-analysis found minimal benefit from adding chemotherapy to treatment in patients over 70, with no survival benefit for those over 70 years old, and potential harm for those over 80 (16). These findings emphasize the importance of appropriate patient selection for CCRT, especially in the elderly.

Given the challenges associated with chemotherapy, particularly in the context of CCRT, efforts have been made to explore treatment de-escalation strategies in select low-risk populations. This has been of particular interest in HPV-associated oropharyngeal cancer, where the favorable prognosis has prompted investigations into reducing treatment intensity while maintaining oncologic efficacy and minimizing toxicity.

One approach to de-escalation has been the use of induction chemotherapy to identify patients eligible for reduced-intensity RT. The ECOG 1308 trial (35) was a phase II study which evaluated this strategy by treating patients with HPV-positive oropharyngeal cancer using induction chemotherapy combined with cetuximab. Patients who achieved CR subsequently received reduced-dose intensity-modulated radiotherapy (IMRT). The study demonstrated favorable PFS and notable improvements in swallowing function and nutritional status. Similarly, a phase II trial by Chen et al. (36) assessed a comparable strategy, where patients with HPV-associated oropharyngeal cancer who demonstrated either CR or partial response (PR) to induction chemotherapy received reduced-dose RT. This approach was associated with high PFS rates and a favorable toxicity profile, further supporting the feasibility of risk-adapted de-escalation.

The OPTIMA trial took this concept further by integrating both response-based and risk-stratified treatment de-intensification (37). In this phase II study, patients with HPV-associated oropharyngeal cancer were treated with induction chemotherapy, followed by dose and volume de-escalation of RT or CCRT based on their response. The trial found that this strategy resulted in favorable oncologic outcomes while reducing both acute and chronic toxicities, reinforcing the potential role of induction chemotherapy in selecting patients for less intensive treatment.

Another notable investigation in this field is the NRG-HN002 trial (38). This phase II study demonstrated that a reduced-intensity CCRT regimen achieved acceptable PFS. However, subsequent data from the NRG-HN005 trial found that this de-escalation approach was not non-inferior in terms of PFS, underscoring the need for caution when applying phase II findings to clinical practice (39). Indeed, a recently published systematic review of clinical trials on treatment de-escalation in HPV-related oropharyngeal cancer recommended against deviating from the standard of care outside of clinical trials (40).

### Practical recommendations for CCRT

CCRT with cisplatin remains the cornerstone treatment, when possible, for improving survival and preserving organ function in LA-HNSCC. However, practical considerations should be applied as we carefully select patients who are fit for this approach. Factors such as performance status, comorbidities and age should be considered in the treatment decisions. For patients over 70, the lack of significant survival benefit from chemotherapy as shown in the MACH-NC meta-analysis, suggests considering non-chemotherapy-based strategies that are forthcoming in this article.

## Immunotherapy and targeted therapy: cisplatin-free alternatives to chemotherapy in LA-HNSCC

Targeted therapy focuses on inhibiting specific molecular pathways critical to cancer cell growth and survival (41). Cetuximab, the only FDA-approved EGFR inhibitor for LA-HNSCC, is used primarily for patients unfit for standard CCRT (42). Trials assessing cetuximab in non-metastatic LA-HNSCC are summarized in **Table 1**. The Bonner et al. trial enrolled 424 patients with LA-HNSCC, who were randomized to receive either RT alone or RT combined with cetuximab (43). Combining cetuximab with RT showed significant improvement in median OS (49.0 months vs. 29.3 months for RT alone; HR 0.73, 95% CI 0.56–0.95; p=0.018). The 5-year OS was 45.6% for the cetuximab plus RT versus 36.4% for the RT-alone. Patients with grade 2 or higher acneiform rash had significantly better survival (HR 0.49, 95% CI 0.34–0.72; p=0.002). However, the control arm did not receive the standard of care therapy, cisplatin. Later trials, including De-ESCALATE (66), ARTSCAN III (67), TROG 12.01 (68), and RTOG 1016 (69), found cisplatin combined with was RT superior to cetuximab plus RT in HPV positive oropharyngeal tumors. Regarding the combination of cetuximab with cisplatin, the RTOG 0522 trial showed that adding cetuximab to cisplatin and RT did not improve OS or PFS (70). Despite these results, cetuximab remains an option for carefully selected patients with LA-HNSCC ineligible for CCRT. Previously, it was questioned whether all subgroups would benefit from cetuximab or only HPV-/p16-positive tumors. However, later it was shown that although p16 and HPV serve as prognostic biomarkers for LA-HNSCC, they have not been shown to predict response to cetuximab-containing regimens in either setting. Therefore, current evidence indicates that the benefits of cetuximab are observed in select patients with either p16-/HPV-positive and -negative HN-SCC (44).

**Table 1:** Key trials which assessed the role of cetuximab in the treatment of HNSCC.

Trial	Population	Methods	RT details	Key Findings
Bonner et al. (43)	Patients with locoregionally advanced oropharyngeal, hypopharyngeal, or laryngeal cancer.	Randomized phase III trial: RT alone vs RT + weekly cetuximab	70 Gy/35 fractions in 7 weeks, or hyperfractionated 72-76.8 Gy/1.2 fractions BID, or with concomitant boost to 72 Gy/42 fractions	Adding cetuximab to RT (without chemotherapy) improved LC (50% vs 41%) and OS (45.6% vs 36.4%)
De-ESCALATE (117)	Patients with low-risk HPV-positive oropharyngeal cancer	Randomized phase III trial: Cetuximab + RT vs. Cisplatin + RT	70 Gy/35 fractions in 7 weeks to both arms	Cetuximab resulted in poorer OS (89% vs 98%) and higher rates of LRR (16% vs 6%) compared to cisplatin.
ARTSCAN III (118)	Patients with locally advanced SCC of the oropharynx, hypopharynx, oral cavity or larynx.	Randomized phase III trial: Cetuximab + RT vs. Cisplatin + RT	70 Gy/35 fractions in 7 weeks, or hyperfractionated 72-76.8 Gy/1.2 fractions BID, or with concomitant boost to 72 Gy/42 fractions	Cumulative incidence of LRF was more than twice as high in the cetuximab arm (23% vs 9%)
TROG 12.01 (119)	Patients with HPV-positive oropharyngeal cancer	Randomized phase III trial: Cetuximab + RT vs. Cisplatin + RT	70 Gy/35 fractions in 7 weeks to both arms	3-year failure-free survival rates were inferior in the cetuximab arm (80% vs 93%)
RTOG 1016 (120)	Patients with HPV-positive oropharyngeal cancer	Randomized phase III trial: Cetuximab + RT vs. Cisplatin + RT	70 Gy/35 fractions in 6 weeks to both arms	Cetuximab resulted in inferior OS (78% vs 85%) and PFS (67% vs 78%) for the cetuximab group compared to the cisplatin group.
RTOG 0522 (121)	Patients with locally advanced oropharyngeal cancer	Randomized phase III trial: Cetuximab + cisplatin + RT vs cisplatin + RT	72 Gy/42 fractions BID or 70 Gy/35 fractions in 6 weeks	The addition of cetuximab did not significantly improve OS (76% vs 73%) or PFS (59% vs 61%)

ARTSCAN, Accelerated Radiotherapy Study in head and neck Cancers; BID, Twice daily; DFS, Disease-free survival; HPV, Human papillomavirus; LRF, Locoregional failure; OS, Overall survival; PFS, Progression-free survival; RT, Radiotherapy; RTOG, Radiation Therapy Oncology Group; SCC, Squamous cell carcinoma; TROG, Trans-Tasman Radiation Oncology Group.

Other anti-EGFR monoclonal antibodies, including panitumumab (45), zalutumumab (46), and nimotuzumab (47), have also been evaluated as concurrent treatments with RT. In a randomized phase III trial for LA-HNSCC, panitumumab failed to show significant improvements in LRC or survival when compared to the standard of care high dose cisplatin with radiation (48).

Immunotherapies, such as immune checkpoint inhibitors (ICIs), enhance the immune system’s ability to target cancer cells by blocking inhibitory pathways (49). Blocking PD-1/PD-L1 signaling with ICIs restores immune function, increasing antitumor activity (50, 51).

The role of immunotherapy in definitive treatment of LA-HNSCC is still unclear. The NRG-HN004 study compared concurrent and adjuvant durvalumab (an anti-PD-L1 antibody) with RT versus, RT with cetuximab, in cisplatin-unfit LA-HNSCC patients (52). Durvalumab did not improve PFS and was associated with worse LRF compared to cetuximab. The NRG-HN005 trial was a phase II/III randomized study targeting patients with p16-positive, non-smoking associated, locoregionally advanced oropharyngeal cancer (39). The study compared two experimental arms with standard 70 Gy RT with cisplatin: 60 Gy RT combined with cisplatin, and 60 Gy RT combined with nivolumab. Results showed that the experimental arms did not meet the non-inferiority criteria; specifically, the 2-year PFS estimates were 98.1% for Arm 1, compared to 88.6% for Arm 2 and 90.3% for Arm 3. The trial’s futility analyses indicated that both experimental arms had significantly poorer outcomes than expected, leading to the conclusion that a phase III trial would not proceed. Similarly, the GORTEC-REACH phase III trial, which evaluated avelumab (anti-PD-L1 antibody) combined with cetuximab and RT versus standard care in LA-HNSCC, found no significant improvement in PFS (53). The JAVELIN Head and Neck 100 (54) phase III trial tested the addition of avelumab to standard CCRT in patients with LA-HNSCC. This trial showed that avelumab did not prolong PFS and was terminated early due to futility. The KEYNOTE-412 phase III trial assessed the addition of pembrolizumab to CCRT in patients with unresected LA-HNSCC. While pembrolizumab combined with CCRT showed a favorable trend towards improved event-free survival (EFS) compared to placebo with CCRT, the difference was not statistically significant. The trial also indicated that patients with higher PD-L1 expression (CPS ≥1 and CPS ≥20) experienced more pronounced benefit (55). Radioimmunotherapy presents a promising and intriguing treatment option for LA-HNSCC. However, further trials are needed to identify specific groups of patients that would benefit from this approach before its application into clinical practice (56, 57).

### Recommendations

Targeted therapies and immunotherapy with RT represent an area of active research for its use in the treatment of LA-HNSCC, which can potentially become an alternative or complementary approach to traditional CCRT. Cetuximab has shown improvement in disease specific outcomes and remains the primary EGFR inhibitor that demonstrated significant efficacy in the treatment of LA-HNSCC, particularly for patients unfit for cisplatin-based CCRT. In patients deemed eligible to receive high dose cisplatin with RT, chemotherapy was found to be superior to cetuximab with RT. The integration of immunotherapy into the treatment of LA-HNSCC remains investigational and its use in this setting should be restricted to clinical trials at this stage. Several factors are expected to impact the outcomes of this approach in LA-HNSCC such as the tumor’s molecular profile and PD-L1 expression.

## Nanoparticle-based therapies: a breakthrough in the treatment of LA-HNSCC

Nanoparticles (NPs), ranging from 1 to 100 nm in size, can carry drugs, imaging agents, and targeting ligands, making them versatile tools in cancer treatment (58). Due to leaky blood vessels and impaired lymphatic drainage in tumors, NPs accumulate in tumor tissues, enhancing targeted delivery and internalization into cancer cells (59, 60). In LA-HNSCC, NPs improve radiosensitivity, enable photothermal therapy, aid immune therapy, and precisely deliver agents to the tumor microenvironment (TME), minimizing damage to healthy tissue and reducing side effects (61).

Gold and silver NPs enhance radiosensitivity by absorbing and scattering radiation energy. Gold NPs, for example, increase reactive oxygen species and enhance DNA damage during RT (62). Cetuximab-targeted gold NPs have shown promising results by enhancing radiation effects, inducing apoptosis, inhibiting angiogenesis, and reducing tumor growth with no toxicity. One study showed these NPs enhanced the radiation effect via earlier and greater apoptosis, diminishing repair mechanisms, and inhibiting angiogenesis, with no observed evidence of toxicity. These NPs also had a significant impact on tumor growth (P < 0.001) (63, 64).

Nanotherapy also alters the TME by targeting immune cells and fibroblasts, regulating angiogenesis and immune responses, and using nanovaccines to activate immunity or modify tumor-associated macrophages for anti-tumor effects (61, 65).

NPs can also deliver chemotherapeutic agents directly to cancer cells, minimizing systemic toxicity. For example, cisplatin-loaded NPs designed to target oral squamous cell carcinoma have shown enhanced intracellular uptake and greater cytotoxic effects (66). The PRV111 trial, a phase I/II clinical study, demonstrated that a NP formulation for local cisplatin delivery resulted in an 87% response rate in patients with advanced oral cavity squamous cell carcinoma. These patients saw a significant reduction in tumor volume, with an average decrease of 70% within a week, and experienced no severe side effects, with no locoregional recurrences over six months (67). Chun et al. examined the efficacy of NAB-paclitaxel, NAB-cisplatin, and NAB-cetuximab in improving the efficacy of RT for LA-HNSCC in a phase I/II trial. At a median follow up of 24 months, LC was 71%, OS was 68%, and PFS was 60% (68). Furthermore, a retrospective analysis revealed the efficacy of NAB-paclitaxel in the treatment of HNSCC that had shown progression after prior use of other taxanes (69).

In ongoing clinical trials, nanoparticle formulations containing paclitaxel, docetaxel, or other drugs are showing promising results in improving cancer treatment. Some patients have experienced substantial tumor shrinkage or even complete remission (70). To achieve effective cancer therapies using NPs, strong interdisciplinary collaboration across various fields is essential, to take these treatments from initial innovation to final intervention.

### Recommendations

Nanoparticle-based therapies hold great potential in the treatment of LA-HNSCC, especially in improving radiosensitivity and targeting the tumor microenvironment. While the early clinical results are promising, challenges such as optimizing nanoparticle delivery, ensuring precise targeting, and overcoming tumor heterogeneity need further attention. Ongoing research should focus on refining nanoparticle formulations, improving their efficacy in combination with existing therapies like chemotherapy and radiotherapy, and developing strategies to reduce potential toxicities. As these therapies move forward, interdisciplinary collaboration will be key to translating nanoparticle-based treatments from preclinical success to widespread clinical application.

## Altered fractionation as an alternative approach

Biological factors such as tumor hypoxia and accelerated repopulation can further limit the effectiveness of conventionally fractionated RT (71). To address these challenges, altered fractionation regimens have been developed. These include accelerated fractionation (AFX) RT, which shortens treatment time to overcome tumor repopulation, and hyperfractionation (HFX) which aims to increase the overall RT dose without increasing late RT toxicities. Both strategies seek to optimize therapeutic outcomes while balancing acute and late side effects (71–78). (For further details on AFX and HFX, please see **Table 2**).

**Table 2:** Comparison of accelerated treatment and hyperfractionation in radiotherapy.

Feature	Accelerated Treatment	Hyperfractionation
**Goal**	Limit tumor cell repopulation by shortening treatment duration.	Increase overall radiation dose with reduction of the dose per fraction to increase local control with reduction of late toxicities.
**Mechanism**	Shortens total treatment time, either maintaining or adjusting dose and fraction size.	Administers smaller doses per fraction (e.g., 1.15-1.2 Gy) to allow higher total doses.
**Strategies**	-Pure accelerated RT: Shortens duration without dose change. - Hybrid accelerated RT: Adjusts dose, fraction size, and time distribution	Increases fraction number while reducing fraction dose. Exploits tumor vs. normal tissue radiosensitivity.
**Therapeutic Advantages**	Reduces opportunity for tumor regeneration during treatment.	Enhances therapeutic ratio by lowering late toxicity risk.
**Impact on Toxicities**	Increased acute toxicities during treatment.	Higher risk of acute toxicities but reduced late toxicities.
**An example of key Trials**	RTOG 9003 (122), RTOG 0129 (123), DAHANCA 6 &7 (124, 125), IAEA-ACC (126)	MARCH (79), RTOG 9003 (122)

DAHANCA, Danish Head and Neck Cancer Study Group; IAEA-ACC, International Atomic Energy Agency Accelerated Radiation Trial; MACH, Meta-Analysis of Chemotherapy in Head and Neck Cancer; MARCH, Meta-Analysis of Radiotherapy in Carcinomas of Head and Neck; RT, Radiotherapy; RTOG, Radiation Therapy Oncology Group.

Bold formatting is used only for the "Feature" column to highlight the comparison criteria.

The role of altered fractionation regimens in HNSCC has been extensively studied, with a detailed summary shown in **Table 3**. The updated MARCH meta-analysis (79), which included 33 trials involving 11,423 patients, reaffirmed the survival benefits of altered fractionation RT over conventional fractionation. Altered fractionation demonstrated a significant OS benefit (HR 0.94; P = 0.0033), with HFX providing the greatest advantage (HR 0.83), resulting in an 8.1% absolute survival improvement at 5 years. However, when altered fractionation RT was compared to CCRT, CCRT demonstrated superior OS outcome (HR 1.22; P = 0.0098). Importantly, among the five trials comparing altered fractionation RT with CCRT included in MARCH meta-analysis, only one utilized HFX, EORTC 22962 which was terminated early, while the other four employed moderately accelerated RT (**Table 4**) (80). These findings establish HFX RT, alongside CCRT, as a standard of care for LA-HNSCC, while highlighting the need for further trials directly comparing these two approaches.

**Table 3:** Key trials on altered fractionation regimens in HNSCC.

Trial	Population	Methods	Key Findings
RTOG 9003 (122)	Patients with locally advanced HNSCC treated with definitive RT	A phase III trial that compared conventionally fractionated RT as 70 Gy/35 fractions, hyperfractionated RT as 81.6 Gy/68 fractions BID, accelerated split-course RT as 67.2 Gy/42 fractions BID, with 2 weeks break at 38.4 Gy, vs accelerated RT with concomitant boost as 72 Gy/42 fractions	Hyperfractionated RT improved OS and LC while reducing late effects compared to conventional RT.
DAHANCA 6 & 7 (124, 125)	Patients with HNSCC treated with definitive RT	Phase III trials that compared six fractions per week of RT (66-68 Gy/33-34 fractions) with nimorazole versus five fractions per week	Six fractions per week resulted in better 5-year LC (70% vs 60%) and DFS (73% vs 66%) compared to five fractions per week.
IAEA-ACC Trial (126)	Patients with HNSCC treated with definitive RT	Phase III trial that compared six fractions per week of RT (66-70 Gy/33-35 fractions) versus five fractions per week	Improved 5-year LC (42% vs 30%) and DFS (50% vs 40%) compared to 5 fractions per week.
RTOG 0129 (123)	Patients with locally advanced HNSCC treated with CCRT	Phase III trial that compared accelerated fractionation with concomitant boost (72 Gy over 6 weeks) and concurrent cisplatin vs. standard fractionation 70 Gy/35 fractions with concurrent cisplatin	No significant differences in 8-year outcomes for OS, PFS or treatment toxicity between the two regimens.
DAHANCA 28 (81)	Patients with HPV-negative locally advanced HNSCC	Phase I/II trial that treated with hyperfractionated accelerated RT (76 Gy/56 fractions BID) with cisplatin and nimorazole	Treatment was feasible, with a 3-year LRF of 21% and OS of 74%. Resulted in acute toxicity in 60% of patients; significant late toxicity observed.
HYPNO (82)	Patients with locally advanced head and neck cancer	Phase III trial that compared hypofractionated RT (55 Gy/20 fractions) vs conventional fractionation (66 Gy in 33 fractions, 6 fractions per week) with optional cisplatin use	Hypofractionated RT was non-inferior to conventional treatment in terms of 3-year LRC (51%), OS (55%), and PFS (45%)

RTOG, Radiation Therapy Oncology Group; MARCH-HN, Meta-Analysis of Radiotherapy in Carcinomas of Head and Neck; DAHANCA, Danish Head and Neck Cancer Study Group; IAEA-ACC, International Atomic Energy Agency Accelerated Radiation Trial; CCRT, Concurrent Chemoradiotherapy; OS, Overall Survival; LC, Local Control; DFS, Disease-Free Survival; BID, Twice a Day; PFS, Progression-Free Survival; LRF, Locoregional failure; HPV, Human Papillomavirus; RT, radiotherapy.

**Table 4:** Trials comparing altered fractionation to CCRT in the MARCH meta-analysis.

Trial Name	Total Patients	Study arms	Key Findings
INRC-HN9 (127)	136	-Alternating chemoradiotherapy, Cisplatin + 5-FU for 5 days on weeks 1,4,7 and 10, alternated with three 2-week courses of RT, 20 Gy per course, 2 Gy per day, 5 days a week -Partly accelerated RT, 70 Gy/40 fractions in six weeks	No difference in outcomes between patients treated with alternating chemoradiotherapy vs those treated with partly accelerated radiotherapy, with the latter group experiencing worse mucosal and skin toxicities.
ORO 93-01 (128)	192	-Conventionally fractionated RT alone, 66-70 Gy/33-35 fractions, 5 days a week -Split-course accelerated hyperfractionated RT, 64-67.2 Gy/40-42 fractions BID, 5 days a week with 2-week split at 38.4 Gy -CCRT, 66-70 Gy/33-35 fractions, 5 days a week, with concurrent carboplatin-5-FU	CCRT is superior to RT alone in terms of improving DFS, but not OS, and was associated with a higher incidence of acute morbidity.
EORTC 22962 (80)	57	2 x 2 design: -70 Gy/35 fractions, 5 days a week vs 80.5 Gy/70 fractions BID, 5 days a week -Concurrent cisplatin vs none	Terminated early due to slow accrual after recruiting only 57 patients.
GORTEC 9902 (18)	840	-CCRT, 70 Gy/35 fractions in 7 weeks, with carboplatin/5-FU -Accelerated CRT, 70 Gy/35 fractions in 6 weeks (2 Gy daily for 40 Gy them 1.5 Gy BID), with carboplatin/5-FU -Very accelerated RT alone, 64.8 Gy/36 fractions BID, in 3.5 weeks	Conventional fractionation with chemotherapy resulted in superior OS and lower toxicity compared to accelerated RT. Acceleration of RT cannot compensate for the absence of chemotherapy.
TMH 1114 (129)	186	-Conventionally fractionated RT alone, 66-70 Gy/33-35 fractions, 5 fractions a week -CCRT, 66-70 Gy/33-35 fractions, 5 days a week, with cisplatin -Accelerated RT alone as 66-70 Gy/33-35 fractions, 6 fractions a week	CCRT showed superior LRC compared to the RT alone arms, with higher but acceptable acute and late toxicities.

RT, Radiotherapy; CCRT, Concurrent Chemoradiotherapy; DFS, Disease-Free Survival; OS, Overall Survival; LRC, Locoregional Control; BID, Twice a Day; 5-FU, 5-Fluorouracil; EORTC, European Organization for Research and Treatment of Cancer; GORTEC, Groupe d’Oncologie Radiothérapie Tête et Cou.

Another important study, DAHANCA 28 (81), investigated the use of nimorazole, a hypoxic radiosensitizer, in combination with hyperfractionated accelerated RT (76 Gy in 56 fractions, administered twice daily) and cisplatin in patients with HPV-negative tumors,. While the trial demonstrated the feasibility of this approach, it also reported high rates of acute toxicity in approximately 60% of patients, along with significant late toxicity.

More recently, the HYPNO study explored hypofractionation in LA-HNSCC (82), delivering higher doses per fraction (>2 Gy/fraction). In this trial, 55 Gy in 20 fractions was non-inferior to conventional fractionation (66 Gy in 33 fractions, administered six fractions per week), with cisplatin use being optional. This finding presents a compelling alternative for patients, providing a shorter treatment course without compromising efficacy.

### Recommendations

Altered fractionation regimens present a valuable approach to improving outcomes in LA-HNSCC, particularly for patients who cannot tolerate chemotherapy. HFX offers the most consistent improvements in OS and local control, but its twice-daily schedule can be difficult to manage. In contrast, hypofractionation, requiring fewer sessions, is more convenient but needs further validation. Treatment should be tailored to each patient’s situation, with HFX ideal for those with reliable access to care and hypofractionation better suited for those with logistical challenges who will not be able to attend a long course (7 weeks) of conventional RT. Phase III RCT comparing HFX to CCRT (with cisplatin) is warranted and represents a real clinical need to better define an effective cisplatin-free approach with HFX.

## The role of FDG-PET in LA-HNSCC

PET-CT scans play a crucial role in the delineation of treatment volumes in LA-HNSCC. They enhance the accuracy of staging by precisely identifying the extent of disease, thereby reducing the risk of geographical misses during radiotherapy (83, 84). This advanced imaging modality helps detect tumor regions or lymph nodes that might be overlooked by conventional imaging techniques. Studies have demonstrated that PET-CT can minimize inter-observer variation in gross tumor volume (GTV) delineation (85, 86) and lead to a reduction in GTV size, potentially decreasing treatment-related toxicity (87). This is particularly important in the era of IMRT, where planning target volumes for setup uncertainty are continuously shrinking (88).

Beyond anatomical delineation, PET-CT also provides valuable insights into tumor biology, identifying areas of hypoxia and high proliferation. This information can guide the use of radiosensitizers, facilitate dose escalation, or inform alternative treatment strategies, ultimately improving the therapeutic ratio and local control. However, despite these advantages, PET-CT is not without limitations. Challenges include its limited spatial resolution, the absence of a standardized method for signal segmentation, and the risk of false-positive results due to inflammation (83, 89–91).

Future research is focused on several key innovations to enhance the clinical utility of PET-CT in HN-SCC. One promising direction is the integration of PET-CT with other imaging modalities or biomarkers, which could further refine treatment planning and enable more personalized therapeutic approaches (92). Another area of advancement lies in the development of novel PET tracers that could provide additional biological insights and improve treatment outcomes (93). Additionally, emerging research in nanomedicine is exploring the potential of nanoparticles to enhance radiation effects and selectively target lymph nodes. These innovations could complement PET-CT-guided radiotherapy, further optimizing treatment strategies for patients with HN-SCC (65).

### Recommendations

PET-CT plays a critical role in the treatment planning of HN-SCC, offering superior accuracy in staging, tumor delineation, and identification of biologically significant tumor regions. Its ability to reduce inter-observer variation, refine target volumes, and provide insights into tumor biology makes it a valuable tool in modern radiotherapy. However, limitations such as spatial resolution constraints, signal segmentation challenges, and the potential for false-positive findings must be addressed to maximize its clinical utility. Future research should focus on improving PET-CT image resolution, developing standardized segmentation methods, and integrating novel tracers that enhance biological characterization. Additionally, combining PET-CT with other imaging modalities, biomarker-driven strategies, or emerging nanotechnologies may further refine treatment personalization and therapeutic outcomes. Until these advancements are realized, PET-CT should be leveraged judiciously within a multidisciplinary framework, ensuring its benefits are maximized while accounting for its current limitations.

## The role of hyperthermia in LA-HNSCC

Hyperthermia has emerged as an adjunctive treatment modality in oncology, offering a promising approach for enhancing the effectiveness of RT in LA-HNSCC. By acting as a potent radiosensitizer, hyperthermia improves tumor control and survival outcomes, particularly in patients unfit for CCRT (94, 95).

Hyperthermia sensitizes cancer cells to RT through multiple means (96). By elevating tissue temperatures to 39°C–45°C, it inhibits DNA repair mechanisms, disrupts tumor vasculature, and enhances oxygenation, thus amplifying the effects of RT (97). Furthermore, it promotes immunogenic cell death and enhances the exposure of tumor antigens, thereby activating anti-tumor immune responses. This dual action not only optimizes local tumor control but also holds potential for systemic cancer management (98).

Combining hyperthermia with RT significantly improves complete response (CR) rates in LA-HNSCC. A meta-analysis by Datta et al. reported a CR rate of 62.5% with thermoradiotherapy compared to 39.6% with RT alone, demonstrating its superior efficacy (99). Hyperthermia also enhances LRC and survival outcomes. In a landmark randomized trial, Valdagni and Amichetti observed improved CR rates in patients with metastatic lymph nodes treated with RT and hyperthermia versus RT alone (82.3% vs 36.8%, P=0.0152), leading to an iso-dose thermal enhancement ratio (TER) of 2.23 (100). Acute local toxicities were also similar between both groups. Similarly, another RCT comparing RT alone to RT combined with hyperthermia showed a statistically significant improvement in CR rates (42.2% vs 78.6%, P<0.05), and in survival rates in the hyperthermia-RT arm, with no dose-limiting toxicity recorded (94). The ESHO 2-85 study, conducted by the European Society for Hyperthermic Oncology, investigated the effectiveness of hyperthermia as an adjunct to RT in treating advanced neck nodes (101). Like previous studies, it revealed that the addition of hyperthermia significantly improved the CR rate, with a notable reduction in the 3-year local failure rate from 68% in the RT-only group to 50% in the RT + hyperthermia group. Thus, incorporating hyperthermia into treatment protocols could provide substantial clinical benefits for patients with advanced neck node involvement in LA-HNSCC.

Despite its promise, hyperthermia faces barriers to widespread adoption. Technical challenges include achieving uniform heat distribution and maintaining precise temperature control. Patient compliance can be hindered by discomfort during treatment sessions. Additionally, the requirement for specialized equipment and trained personnel limits its availability in many clinical settings. However, while it is not yet standard practice, it is a consideration in some patients, particularly those unable to tolerate chemotherapy. Future clinical trials should focus on optimizing the integration of thermoradiotherapy in the multimodal treatment options of LA-HNSCC (102).

### Recommendations

Hyperthermia represents a compelling adjunct treatment in the management of LA-HNSCC, particularly in patients unable to tolerate standard CCRT. It has shown the ability to enhance radiosensitivity, improve tumor oxygenation, and boost local control and survival rates. Despite its promise, barriers such as technical challenges, limited availability, and patient compliance must be addressed to facilitate broader adoption. Future research should prioritize optimizing hyperthermia delivery techniques, refining patient selection criteria, and exploring its integration with advanced treatment modalities, including immunotherapy. Until then, hyperthermia should be considered on a case-by-case basis within a personalized treatment framework, particularly for patients with limited therapeutic options.

## Photodynamic therapy in head and neck cancer

Photodynamic therapy (PDT) is a minimally invasive treatment that combines a photosensitizing agent with light exposure to target and destroy cancerous cells. This technique relies on three key components: a photosensitizer, light, and oxygen. When the photosensitizer is activated by light, it generates reactive oxygen species (ROS), which lead to cell death and can also disrupt blood vessels feeding the tumor, thereby limiting its oxygen supply (103, 104).

PDT has shown promise in the management of LA-HNSCC, and has been suggested to be as effective as conventional therapies for the treatment of early-stage HNSCC (105). A retrospective study was conducted to evaluate the outcomes of temoporfin-mediated PDT in patients with functionally inoperable oral or oropharyngeal squamous cell carcinoma. PDT achieved CR in 76.9% of cases, with recurrence-free rates of 60.6%, 48.5%, and 32.3% at six months, one year, and two years, respectively. The treatment maintained swallowing and airway functionality in most patients, demonstrating durable local control in a subset of patients with an acceptable toxicity profile (106). In early and pre-malignant lesions of the head and neck, a phase I trial found that the use of PDT resulted in a 69% CR rate at three months, with the treatment being generally well-tolerated (107). One study on the use of porfimer sodium-mediated PDT in HNSCC has shown that its use had an efficacy rate of 97%, and lead to a CR rate of 72.7% (108). Another study found its use associated with a 5-year OS of 57.8%, with improved quality of life in patients with recurrent or residual disease (109).

PDT offers several advantages over traditional treatment methods, such as surgery and chemoradiation. Since photosensitizers require light activation, they inherently have low systemic toxicity. Additionally, the photochemical reaction is non-thermal, which helps minimize PDT-related morbidity and disfigurement compared to conventional treatments (103). However, PDT faces several limitations that restrict its broader clinical application (110). The reliance on light for activation of the photosensitizer is a central challenge. While this feature allows for spatial control of therapy, it significantly limits PDT’s efficacy in treating deep-seated tumors (111). Another major limitation is the dependency of PDT on oxygen for generating ROS. Many tumors are hypoxic due to their rapid growth and inadequate vasculature, which reduces the effectiveness of PDT (112). Additionally, PDT itself can deplete local oxygen levels, further inhibiting its efficacy (113). Addressing these limitations through technological advancements and tailored treatment protocols will be key to expanding PDT’s utility in oncology.

### Recommendations

PDT offers a promising, minimally invasive option for LA-HNSCC, with advantages like low systemic toxicity and reduced morbidity. However, its efficacy is limited by challenges such as poor light penetration and tumor hypoxia. Advancements in light delivery systems, oxygen-enhancing strategies, and the development of oxygen-independent photosensitizers are needed to address these limitations. Future efforts should also focus on combining PDT with other modalities like chemotherapy or immunotherapy to enhance outcomes. Interdisciplinary collaboration will be key to expanding PDT’s clinical utility and improving patient care.

## Future directions

The evolving treatment landscape for LA-HNSCC emphasizes innovation, balancing efficacy with toxicity, and exploring novel regimens and addressing unmet needs.

The potential of maintenance immunotherapy is being explored in the ECOG-EA3161 trial (NCT03811015), which assesses whether maintenance nivolumab after definitive treatment with RT and cisplatin improves survival outcomes for intermediate-risk HPV-positive oropharyngeal cancer (114). Additionally, a study by the Canadian Cancer Trials Group (NCT03410615) is examining combinations of durvalumab, tremelimumab, and RT in intermediate-risk HPV-positive locoregionally advanced oropharyngeal squamous cell carcinoma (115).

Hyperfractionation (HFX) enhances survival in cisplatin-ineligible patients but poses challenges with toxicity and logistics (79). Future studies should compare HFX and hypofractionation, focusing on clinical outcomes, side effects, logistical feasibility, and cost-effectiveness.

Targeted therapy and immunotherapy combined with RT continue to demonstrate potential for improved outcomes. Cetuximab remains an option for patients intolerant to cisplatin, but its role compared to CCRT requires further validation (43). Trials like NRG HN004 (52) contribute to this understanding, but the role for other trials examining different agents in cisplatin-ineligible patients remains unclear.

Nanoparticle-based therapies represent a cutting-edge frontier in LA-HNSCC treatment. A notable example is the Phase III study of NBTXR3 (NCT04892173), which evaluates this novel nanoparticle agent in platinum-based chemotherapy-ineligible elderly patients with LA-HNSCC (116). In this trial, participants are treated with RT alone or RT in combination with cetuximab, based on the investigator’s choice, and are randomized to receive either standard treatment or NBTXR3. Participants in both arms receive 70 Gy in 35 fractions over seven weeks. The trial aims to assess whether NBTXR3 enhances the efficacy of RT, potentially offering a transformative option for patients unable to tolerate systemic chemotherapy.

## Conclusions

The treatment landscape for LA-HNSCC is progressing towards less toxic, chemotherapy-free alternatives. Altered fractionation regimens, targeted therapies such as EGFR inhibitors, and emerging radioimmunotherapy approaches offer potential options, particularly for patients unable to tolerate chemotherapy. Hyperfractionated RT alone was never compared to CRT with high dose cisplatin in a phase III RCT despite the highest level of evidence for both by meta-analyses of RCT (MACH-NC and MARCH). Further clinical trials are needed to explore this question in greater depth.

## References

[1] Sung H Ferlay J Siegel RL Laversanne M Soerjomataram I Jemal A. Global cancer statistics 2020: GLOBOCAN estimates of incidence and mortality worldwide for 36 cancers in 185 countries. CA Cancer J Clin. (2021) 71:209–49. doi: 10.3322/caac.21660 33538338

[2] Zhou T Huang W Wang X Zhang J Zhou E Tu Y. Global burden of head and neck cancers from 1990 to 2019. iScience. (2024) 27:109282. doi: 10.1016/j.isci.2024.109282 38455975 PMC10918270

[3] Mahal BA Catalano PJ Haddad RI Hanna GJ Kass JI Schoenfeld JD. Incidence and demographic burden of HPV-associated oropharyngeal head and neck cancers in the United States. Cancer Epidemiol Biomarkers Prev. (2019) 28:1660–7. doi: 10.1158/1055-9965.EPI-19-0038 31358520

[4] Barsouk A Aluru JS Rawla P Saginala K Barsouk A. Epidemiology, risk factors, and prevention of head and neck squamous cell carcinoma. Med Sci. (2023) 11:42. doi: 10.3390/medsci11020042 PMC10304137 37367741

[5] Mohamad I Glaun MDE Prabhash K Busheri A Lai SY Noronha V. Current treatment strategies and risk stratification for oral carcinoma. Am Soc Clin Oncol Educ Book. (2023) 43:e389810. doi: 10.1200/EDBK_389810 37200591

[6] Forastiere AA Zhang Q Weber RS Maor MH Goepfert H Pajak TF. Long-term results of RTOG 91-11: A comparison of three nonsurgical treatment strategies to preserve the larynx in patients with locally advanced larynx cancer. J Clin Oncol. (2013) 31:845–52. doi: 10.1200/JCO.2012.43.6097 PMC3577950 23182993

[7] Oun R Moussa YE Wheate NJ. The side effects of platinum-based chemotherapy drugs: A review for chemists. Dalton Trans. (2018) 47:6645–53. doi: 10.1039/C8DT00838H 29632935

[8] Wang H Zheng Z Zhang Y Bian C Bao J Xin Y. Locally advanced head and neck squamous cell carcinoma treatment efficacy and safety: A systematic review and network meta-analysis. Front Pharmacol. (2023) 14:1269863. doi: 10.3389/fphar.2023.1269863 37795033 PMC10546034

[9] Leeman JE Li J Pei X Venigalla P Zumsteg ZS Katsoulakis E. Patterns of treatment failure and postrecurrence outcomes among patients with locally advanced head and neck squamous cell carcinoma after chemoradiotherapy using modern radiation techniques. JAMA Oncol. (2017) 3:1487–94. doi: 10.1001/jamaoncol.2017.0973 PMC5710194 28542679

[10] Bøje CR. Impact of comorbidity on treatment outcome in head and neck squamous cell carcinoma – A systematic review. Radiother Oncol. (2014) 110:81–90. doi: 10.1016/j.radonc.2013.07.005 23953753

[11] Dickstein DR Lehrer EJ Hsieh K Hotca A Jones BM Powers A. Management of older adults with locally advanced head and neck cancer. Cancers. (2022) 14:2809. doi: 10.3390/cancers14112809 35681789 PMC9179912

[12] Beynon RA Lang S Schimansky S Penfold CM Waylen A Thomas SJ. Tobacco smoking and alcohol drinking at diagnosis of head and neck cancer and all-cause mortality: results from head and neck 5000, a prospective observational cohort of people with head and neck cancer. Int J Cancer. (2018) 143:1114–27. doi: 10.1002/ijc.31416 PMC6099366 29607493

[13] Savvides P. (Panos) the role of chemotherapy in the management of patients with head and neck cancer. Semin Plast Surg. (2010) 24:137–47. doi: 10.1055/s-0030-1255331 PMC3324245 22550434

[14] Mueller S Schittenhelm M Honecker F Malenke E Lauber K Wesselborg S. Cell-cycle progression and response of germ cell tumors to cisplatin *in vitro* . Int J Oncol. (2006) 29:471–9. doi: 10.3892/ijo.29.2.471 16820891

[15] Boeckman HJ Trego KS Henkels KM Turchi JJ. Cisplatin sensitizes cancer cells to ionizing radiation via inhibition of non-homologous end joining. Mol Cancer Res MCR. (2005) 3:277–85. doi: 10.1158/1541-7786.MCR-04-0032 PMC2432110 15886299

[16] Lacas B Carmel A Landais C Wong SJ Licitra L Tobias JS. Meta-analysis of chemotherapy in head and neck cancer (MACH-NC): an update on 107 randomized trials and 19,805 patients, on behalf of MACH-NC group. Radiother Oncol. (2021) 156:281–93. doi: 10.1016/j.radonc.2021.01.013 PMC8386522 33515668

[17] Adelstein DJ Li Y Adams GL Wagner H Kish JA Ensley JF. An intergroup phase III comparison of standard radiation therapy and two schedules of concurrent chemoradiotherapy in patients with unresectable squamous cell head and neck cancer. J Clin Oncol. (2003) 21:92–8. doi: 10.1200/JCO.2003.01.008 12506176

[18] Bourhis J Sire C Graff P Grégoire V Maingon P Calais G. Concomitant Chemoradiotherapy versus Acceleration of Radiotherapy with or without Concomitant Chemotherapy in Locally Advanced Head and Neck Carcinoma (GORTEC 99-02): An Open-Label Phase 3 Randomised Trial. Lancet Oncol. (2012) 13:145–53. doi: 10.1016/S1470-2045(11)70346-1 22261362

[19] Denis F Garaud P Bardet E Alfonsi M Sire C Germain T. Final results of the 94-01 french head and neck oncology and radiotherapy group randomized trial comparing radiotherapy alone with concomitant radiochemotherapy in advanced-stage oropharynx carcinoma. J Clin Oncol. (2004) 22:69–76. doi: 10.1200/JCO.2004.08.021 14657228

[20] Medina JA Rueda A de Pasos AS Contreras J Cobo M Moreno P. Phase II study of concomitant boost radiation plus concurrent weekly cisplatin for locally advanced unresectable head and neck carcinomas. Radiother Oncol J Eur Soc Ther Radiol Oncol. (2006) 79:34–8. doi: 10.1016/j.radonc.2006.03.010 16626826

[21] Beckmann GK Hoppe F Pfreundner L Flentje MP. Hyperfractionated accelerated radiotherapy in combination with weekly cisplatin for locally advanced head and neck cancer. Head Neck. (2005) 27:36–43. doi: 10.1002/hed.20111 15459918

[22] Hoek J Bloemendal KM van der Velden L-AA van Diessen JNA van Werkhoven E Klop WMC. Nephrotoxicity as a dose-limiting factor in a high-dose cisplatin-based chemoradiotherapy regimen for head and neck carcinomas. Cancers. (2016) 8:21. doi: 10.3390/cancers8020021 26891330 PMC4773744

[23] Rademaker-Lakhai JM Crul M Zuur L Baas P Beijnen JH Simis YJW. Relationship between cisplatin administration and the development of ototoxicity. J Clin Oncol Off J Am Soc Clin Oncol. (2006) 24:918–24. doi: 10.1200/JCO.2006.10.077 16484702

[24] Chattaraj A Syed MP Low CA Owonikoko TK. Cisplatin-induced ototoxicity: A concise review of the burden, prevention, and interception strategies. JCO Oncol Pract. (2023) 19(5):278–83. doi: 10.1200/OP.22.00710 PMC10414722 36921239

[25] Staff NP Cavaletti G Islam B Lustberg M Psimaras D Tamburin S. Platinum-induced peripheral neurotoxicity: from pathogenesis to treatment. J Peripher Nerv Syst JPNS. (2019) 24:S26–39. doi: 10.1111/jns.12335 PMC6818741 31647151

[26] Cavaletti G. Peripheral neurotoxicity of platinum-based chemotherapy. Nat Rev Cancer. (2008) 8:72–2. doi: 10.1038/nrc2167-c1 18159632

[27] Hamauchi S Yokota T Onozawa Y Ogawa H Onoe T Kamijo T. Safety and efficacy of concurrent carboplatin plus radiotherapy for locally advanced head and neck cancer patients ineligible for treatment with cisplatin. Jpn J Clin Oncol. (2015) 45:1116–21. doi: 10.1093/jjco/hyv142 26423341

[28] Singh K Singh E Rana MK Sharma P Sachdeva S. Adherence to radiotherapy in the treatment of cancer patients: A tertiary care institute experience at punjab. Asian Pac. J Cancer Care. (2022) 7:3–8. doi: 10.31557/apjcc.2022.7.1.3-8

[29] Iqbal MS Chaw C Kovarik J Aslam S Jackson A Kelly J. Primary concurrent chemoradiation in head and neck cancers with weekly cisplatin chemotherapy: analysis of compliance, toxicity and survival. Int Arch Otorhinolaryngol. (2017) 21:171–7. doi: 10.1055/s-0036-1594020 PMC5375948 28382126

[30] Dronkers EAC Mes SW Wieringa MH van der Schroeff MP Baatenburg de Jong RJ. Noncompliance to guidelines in head and neck cancer treatment; associated factors for both patient and physician. BMC Cancer. (2015) 15:515. doi: 10.1186/s12885-015-1523-3 26163015 PMC4499219

[31] Miller JL Evers J. Barriers to adherence to cancer treatments among head and neck cancer patients. J Adv Pract Oncol. (2022) 13:515–23. doi: 10.6004/jadpro.2022.13.5.5 PMC9328454 35910497

[32] Park JW Roh J-L Lee S-W Kim S-B Choi S-H Nam SY. Effect of polypharmacy and potentially inappropriate medications on treatment and posttreatment courses in elderly patients with head and neck cancer. J Cancer Res Clin Oncol. (2016) 142:1031–40. doi: 10.1007/s00432-015-2108-x PMC11819017 26744323

[33] Mohamed MR Ramsdale E Loh KP Arastu A Xu H Obrecht S. Associations of polypharmacy and inappropriate medications with adverse outcomes in older adults with cancer: A systematic review and meta-analysis. Oncol. (2020) 25:e94–e108. doi: 10.1634/theoncologist.2019-0406 PMC6964156 31570516

[34] Machtay M Moughan J Trotti A Garden AS Weber RS Cooper JS. Factors associated with severe late toxicity after concurrent chemoradiation for locally advanced head and neck cancer: an RTOG analysis. J Clin Oncol. (2008) 26:3582–9. doi: 10.1200/JCO.2007.14.8841 PMC4911537 18559875

[35] Marur S Li S Cmelak AJ Gillison ML Zhao WJ Ferris RL. E1308: phase II trial of induction chemotherapy followed by reduced-dose radiation and weekly cetuximab in patients with HPV-associated resectable squamous cell carcinoma of the oropharynx- ECOG-ACRIN cancer research group. J Clin Oncol. (2017) 35:490–7. doi: 10.1200/JCO.2016.68.3300 PMC5455313 28029303

[36] Chen AM Felix C Wang P-C Hsu S Basehart V Garst J. Reduced-dose radiotherapy for human papillomavirus-associated squamous-cell carcinoma of the oropharynx: A single-arm, phase 2 study. Lancet Oncol. (2017) 18:803–11. doi: 10.1016/S1470-2045(17)30246-2 PMC6488353 28434660

[37] Seiwert TY Foster CC Blair EA Karrison TG Agrawal N Melotek JM. OPTIMA: A phase II dose and volume de-escalation trial for human papillomavirus-positive oropharyngeal cancer. Ann Oncol. (2019) 30:297–302. doi: 10.1093/annonc/mdy522 30481287

[38] Yom SS Torres-Saavedra P Caudell JJ Waldron JN Gillison ML Xia P. Reduced-dose radiation therapy for HPV-associated oropharyngeal carcinoma (NRG oncology HN002). J Clin Oncol. (2021) 39:956–65. doi: 10.1200/JCO.20.03128 PMC8078254 33507809

[39] Yom SS Harris J Caudell JJ Geiger JL Waldron J Gillison M. Interim futility results of NRG-HN005, A randomized, phase II/III non-inferiority trial for non-smoking P16+ Oropharyngeal cancer patients. Int J Radiat Oncol Biol Phys. (2024) 120:S2–3. doi: 10.1016/j.ijrobp.2024.08.014

[40] Iorio GC Arcadipane F Martini S Ricardi U Franco P. Decreasing treatment burden in HPV-related OPSCC: A systematic review of clinical trials. Crit Rev Oncol Hematol. (2021) 160:103243. doi: 10.1016/j.critrevonc.2021.103243 33516806

[41] Li Q Tie Y Alu A Ma X Shi H. Targeted therapy for head and neck cancer: signaling pathways and clinical studies. Signal Transduction Targeting Ther. (2023) 8:31. doi: 10.1038/s41392-022-01297-0 PMC9842704 36646686

[42] Taberna M Oliva M Mesía R. Cetuximab-containing combinations in locally advanced and recurrent or metastatic head and neck squamous cell carcinoma. Front Oncol. (2019) 9:383. doi: 10.3389/fonc.2019.00383 31165040 PMC6536039

[43] Bonner JA Harari PM Giralt J Azarnia N Shin DM Cohen RB. Radiotherapy plus cetuximab for squamous-cell carcinoma of the head and neck. N Engl J Med. (2006) 354:567–78. doi: 10.1056/NEJMoa053422 16467544

[44] Bonner JA Mesia R Giralt J Psyrri A Keilholz U Rosenthal DI. P16, HPV, and cetuximab: what is the evidence? Oncol. (2017) 22:811–22. doi: 10.1634/theoncologist.2016-0433 PMC5507644 28526718

[45] Giralt J Trigo J Nuyts S Ozsahin M Skladowski K Hatoum G. Panitumumab plus Radiotherapy versus Chemoradiotherapy in Patients with Unresected, Locally Advanced Squamous-Cell Carcinoma of the Head and Neck (CONCERT-2): A Randomised, Controlled, Open-Label Phase 2 Trial. Lancet Oncol. (2015) 16:221–32. doi: 10.1016/S1470-2045(14)71200-8 25596659

[46] Schick U Gujral DM Richards TM Harrington KJ Nutting CM. Zalutumumab in head and neck cancer. Expert Opin Biol Ther. (2012) 12:119–25. doi: 10.1517/14712598.2012.643864 22171666

[47] Reddy BKM Lokesh V Vidyasagar MS Shenoy K Babu KG Shenoy A. Nimotuzumab provides survival benefit to patients with inoperable advanced squamous cell carcinoma of the head and neck: A randomized, open-label, phase IIb, 5-year study in Indian patients. Oncol. (2014) 50:498–505. doi: 10.1016/j.oraloncology.2013.11.008 24613543

[48] Siu LL Waldron JN Chen BE Winquist E Wright JR Nabid A. Effect of standard radiotherapy with cisplatin vs accelerated radiotherapy with panitumumab in locoregionally advanced squamous cell head and neck carcinoma: A randomized clinical trial. JAMA Oncol. (2017) 3:220–6. doi: 10.1001/jamaoncol.2016.4510 27930762

[49] Wang C-W Biswas PK Islam A Chen M-K Chueh PJ. The use of immune regulation in treating head and neck squamous cell carcinoma (HNSCC). Cells. (2024) 13:413. doi: 10.3390/cells13050413 38474377 PMC10930979

[50] Ferris RL. Immunology and immunotherapy of head and neck cancer. J Clin Oncol. (2015) 33:3293–304. doi: 10.1200/JCO.2015.61.1509 PMC4586169 26351330

[51] Farkona S Diamandis EP Blasutig IM. Cancer immunotherapy: the beginning of the end of cancer? BMC Med. (2016) 14:73. doi: 10.1186/s12916-016-0623-5 27151159 PMC4858828

[52] Mell LK Torres-Saavedra P Wong S Chang S Kish JA Minn AJ. Radiotherapy with durvalumab vs. Cetuximab in patients with locoregionally advanced head and neck cancer and a contraindication to cisplatin: phase II results of NRG-HN004. Int J Radiat Oncol Biol Phys. (2022) 114:1058. doi: 10.1016/j.ijrobp.2022.09.003

[53] Bourhis J Tao Y Sun X Sire C Martin L Liem X. LBA35 avelumab-cetuximab-radiotherapy versus standards of care in patients with locally advanced squamous cell carcinoma of head and neck (LA-SCCHN): randomized phase III GORTEC-REACH trial. Ann Oncol. (2021) 32:S1310. doi: 10.1016/j.annonc.2021.08.2112

[54] Lee NY Ferris RL Psyrri A Haddad RI Tahara M Bourhis J. Avelumab plus Standard-of-Care Chemoradiotherapy versus Chemoradiotherapy Alone in Patients with Locally Advanced Squamous Cell Carcinoma of the Head and Neck: A Randomised, Double-Blind, Placebo-Controlled, Multicentre, Phase 3 Trial. Lancet Oncol. (2021) 22:450–62. doi: 10.1016/S1470-2045(20)30737-3 33794205

[55] Machiels J-P Tao Y Burtness B Tahara M Rischin D Alves GV. LBA5 Primary Results of the Phase III KEYNOTE-412 Study: Pembrolizumab (Pembro) with Chemoradiation Therapy (CRT) vs Placebo plus CRT for Locally Advanced (LA) Head and Neck Squamous Cell Carcinoma (HNSCC). Ann Oncol. (2022) 33:S1399. doi: 10.1016/j.annonc.2022.08.029

[56] Study details | Quad shot Radiotherapy in Combination With Immune Checkpoint Inhibition | ClinicalTrials.Gov . Available online at: https://clinicaltrials.gov/study/NCT04454489 (Accessed 29 September 2024).

[57] Study details | Combining immunotherapy salvage surgery & IORT tx persistent/recurrent head & Neck cancer | ClinicalTrials.Gov . Available online at: https://clinicaltrials.gov/study/NCT04754321 (Accessed 30 September 2024).

[58] Wu T-T Zhou S-H. Nanoparticle-based targeted therapeutics in head-and-neck cancer. Int J Med Sci. (2015) 12:187–200. doi: 10.7150/ijms.10083 25589895 PMC4293184

[59] Dvorak HF. Leaky tumor vessels: consequences for tumor stroma generation and for solid tumor therapy. Prog Clin Biol Res. (1990) 354A:317–30. 2247496

[60] Kobayashi H Watanabe R Choyke PL. Improving conventional enhanced permeability and retention (EPR) effects; what is the appropriate target? Theranostics. (2013) 4:81–9. doi: 10.7150/thno.7193 PMC3881228 24396516

[61] Zhang Y Dong P Yang L. The role of nanotherapy in head and neck squamous cell carcinoma by targeting tumor microenvironment. Front Immunol. (2023) 14:1189323. doi: 10.3389/fimmu.2023.1189323 37292204 PMC10244756

[62] Chen Y Yang J Fu S Wu J. Gold nanoparticles as radiosensitizers in cancer radiotherapy. Int J Nanomed. (2020) 15:9407–30. doi: 10.2147/IJN.S272902 PMC7699443 33262595

[63] Popovtzer A Mizrachi A Motiei M Bragilovski D Lubimov L Levi M. Actively targeted gold nanoparticles as novel radiosensitizer agents: an *in vivo* head and neck cancer model. Nanoscale. (2016) 8:2678–85. doi: 10.1039/C5NR07496G 26757746

[64] Gargioni E Schulz F Raabe A Burdak-Rothkamm S Rieckmann T Rothkamm K. Targeted nanoparticles for tumour radiotherapy enhancement—the long dawn of a golden era? Ann Transl Med. (2016) 4:523–3. doi: 10.21037/atm.2016.12.46 PMC5233477 28151534

[65] Li H-X Gong Y-W Yan P-J Xu Y Qin G Wen W-P. Revolutionizing head and neck squamous cell carcinoma treatment with nanomedicine in the era of immunotherapy. Front Immunol. (2024) 15. doi: 10.3389/fimmu.2024.1453753 PMC11638222 39676875

[66] Wang Z-Q Liu K Huo Z-J Li X-C Wang M Liu P. Cell-targeted chemotherapeutic nanomedicine strategy for oral squamous cell carcinoma therapy. J Nanobiotechnol. (2015) 13:63. doi: 10.1186/s12951-015-0116-2 PMC4591064 26427800

[67] Goldberg M Manzi A Birdi A Laporte B Conway P Cantin S. A nanoengineered topical transmucosal cisplatin delivery system induces anti-tumor response in animal models and patients with oral cancer. Nat Commun. (2022) 13:4829. doi: 10.1038/s41467-022-31859-3 35977936 PMC9385702

[68] Chun SG Hughes R Sumer BD Myers LL Truelson JM Khan SA. A phase I/II study of nab-paclitaxel, cisplatin, and cetuximab with concurrent radiation therapy for locally advanced squamous cell cancer of the head and neck. Cancer Invest. (2017) 35:23–31. doi: 10.1080/07357907.2016.1213275 27892728

[69] Ley J Wildes TM Daly K Oppelt P Adkins D. Clinical benefit of nanoparticle albumin-bound-paclitaxel in recurrent/metastatic head and neck squamous cell carcinoma resistant to cremophor-based paclitaxel or docetaxel. Med Oncol. (2017) 34:28. doi: 10.1007/s12032-017-0884-7 28078561 PMC6754095

[70] Chavda VP Balar PC Patel SB. Nanotheranostics-based management of head and neck cancer. Nanotheranostics. (2023) 7:202–9. doi: 10.7150/ntno.81724 PMC9925351 36793352

[71] Withers HR Taylor JM Maciejewski B. The hazard of accelerated tumor clonogen repopulation during radiotherapy. Acta Oncol Stockh Swed. (1988) 27:131–46. doi: 10.3109/02841868809090333 3390344

[72] Marcu LG Dasu IT Dasu A Marcu LG Dasu IT Dasu A. The six rs of head and neck cancer radiotherapy. In: Contemporary Issues in Head and Neck Cancer Management. IntechOpen (2015).

[73] Nguyen LN Ang KK. Radiotherapy for cancer of the head and neck: altered fractionation regimens. Lancet Oncol. (2002) 3:693–701. doi: 10.1016/S1470-2045(02)00906-3 12424072

[74] Dische S Saunders MI. Continuous, hyperfractionated, accelerated radiotherapy (CHART). British J Can. (1989) 59:325–6. 10.1038/bjc.1989.64 PMC2247093 2649133

[75] Matuschek C Haussmann J Bölke E Gripp S Schuler PJ Tamaskovics B. Accelerated vs. Conventionally fractionated adjuvant radiotherapy in high-risk head and neck cancer: A meta-analysis. Radiat Oncol Lond Engl. (2018) 13:195. doi: 10.1186/s13014-018-1133-8 PMC6172789 30286777

[76] Isobe K Uno T Kawakami H Ueno N Aruga T Yasuda S. Hyperfractionated radiation therapy for locoregionally advanced nasopharyngeal cancer. Jpn J Clin Oncol. (2005) 35:116–20. doi: 10.1093/jjco/hyi041 15741300

[77] Ghaderi N Jung J Brüningk SC Subramanian A Nassour L Peacock JA. Century of fractionated radiotherapy: how mathematical oncology can break the rules. Int J Mol Sci. (2022) 23:1316. doi: 10.3390/ijms23031316 35163240 PMC8836217

[78] Akimoto T. Radiobiological basis for hyperfractionated radiation therapy. Gan To Kagaku Ryoho. (2008) 35:1820–2. 19011328

[79] Lacas B Bourhis J Overgaard J Zhang Q Grégoire V Nankivell M. Role of radiotherapy fractionation in head and neck cancers (MARCH): an updated meta-analysis. Lancet Oncol. (2017) 18:1221–37. doi: 10.1016/S1470-2045(17)30458-8 PMC5737765 28757375

[80] Clinical Trials Database . Available online at: https://www.eortc.org/research_field/clinical-detail/22962/ (Accessed 14 December 2024).

[81] Saksø M Jensen K Andersen M Hansen CR Eriksen JG Overgaard J. DAHANCA 28: A phase I/II feasibility study of hyperfractionated, accelerated radiotherapy with concomitant cisplatin and nimorazole (HART-CN) for patients with locally advanced, HPV/P16-negative squamous cell carcinoma of the oropharynx, hypopharynx, larynx and oral cavity. Radiother Oncol. (2020) 148:65–72. doi: 10.1016/j.radonc.2020.03.025 32335364

[82] Bentzen SM Rosenblatt E Gupta T Agarwal JP Laskar SG Bhasker S. Randomized Controlled Trial of Hypofractionated vs. Normo-Fractionated Accelerated Radiation Therapy with or without Cisplatin for Locally Advanced Head and Neck Squamous Cell Carcinoma (HYPNO). Int J Radiat Oncol Biol Phys. (2023) 117:e2. doi: 10.1016/j.ijrobp.2023.08.026 41711624

[83] Newbold K Powell C. PET/CT in radiotherapy planning for head and neck cancer. Front Oncol. (2012) 2:189. doi: 10.3389/fonc.2012.00189 23233906 PMC3518254

[84] Goel R Moore W Sumer B Khan S Sher D Subramaniam RM. Clinical practice in PET/CT for the management of head and neck squamous cell cancer. Am J Roentgenol. (2017) 209:289–303. doi: 10.2214/AJR.17.18301 28731808

[85] Ciernik IF Dizendorf E Baumert BG Reiner B Burger C Davis JB. Radiation treatment planning with an integrated positron emission and computer tomography (PET/CT): A feasibility study. Int J Radiat Oncol Biol Phys. (2003) 57:853–63. doi: 10.1016/s0360-3016(03)00346-8 14529793

[86] Riegel AC Berson AM Destian S Ng T Tena LB Mitnick RJ. Variability of gross tumor volume delineation in head-and-neck cancer using CT and PET/CT fusion. Int J Radiat Oncol Biol Phys. (2006) 65:726–32. doi: 10.1016/j.ijrobp.2006.01.014 16626888

[87] Daisne J-F Duprez T Weynand B Lonneux M Hamoir M Reychler H. Tumor volume in pharyngolaryngeal squamous cell carcinoma: comparison at CT, MR imaging, and FDG PET and validation with surgical specimen. Radiology. (2004) 233:93–100. doi: 10.1148/radiol.2331030660 15317953

[88] Madani I Duthoy W Derie C De Gersem W Boterberg T Saerens M. Positron emission tomography-guided, focal-dose escalation using intensity-modulated radiotherapy for head and neck cancer. Int J Radiat Oncol Biol Phys. (2007) 68:126–35. doi: 10.1016/j.ijrobp.2006.12.070 17448871

[89] Thorwarth D Eschmann S-M Paulsen F Alber M. Hypoxia dose painting by numbers: A planning study. Int J Radiat Oncol Biol Phys. (2007) 68:291–300. doi: 10.1016/j.ijrobp.2006.11.061 17448882

[90] Lee NY Mechalakos JG Nehmeh S Lin Z Squire OD Cai S. Fluorine-18-labeled fluoromisonidazole positron emission and computed tomography-guided intensity-modulated radiotherapy for head and neck cancer: A feasibility study. Int J Radiat Oncol Biol Phys. (2008) 70:2–13. doi: 10.1016/j.ijrobp.2007.06.039 17869020 PMC2888477

[91] Rajendran J Krohn K. F18 fluoromisonidazole for imaging tumor hypoxia: imaging the microenvironment for personalized cancer therapy. Semin Nucl Med. (2015) 45:151–62. doi: 10.1053/j.semnuclmed.2014.10.006 PMC4339212 25704387

[92] Paidpally V Chirindel A Lam S Agrawal N Quon H Subramaniam RM. FDG-PET/CT imaging biomarkers in head and neck squamous cell carcinoma. Imaging Med. (2012) 4:633–47. doi: 10.2217/iim.12.60 PMC3587845 23482696

[93] Mason C Gimblet GR Lapi SE Lewis JS. Novel tracers and radionuclides in PET imaging. Radiol Clin North Am. (2021) 59:887–918. doi: 10.1016/j.rcl.2021.05.012 34392925 PMC9116257

[94] Huilgol NG Gupta S Sridhar CR. Hyperthermia with radiation in the treatment of locally advanced head and neck cancer: A report of randomized trial. J Cancer Res Ther. (2010) 6:492–6. doi: 10.4103/0973-1482.77101 21358087

[95] Hua Y Ma S Fu Z Hu Q Wang L Piao Y. Intracavity hyperthermia in nasopharyngeal cancer: A phase III clinical study. Int J Hyperth. (2011) 27:180–6. doi: 10.3109/02656736.2010.503982 20858104

[96] Datta NR Bodis S. Hyperthermia with radiotherapy reduces tumour alpha/beta: insights from trials of thermoradiotherapy vs radiotherapy alone. Radiother Oncol. (2019) 138:1–8. doi: 10.1016/j.radonc.2019.05.002 31132683

[97] Vujaskovic Z Song CW. Physiological mechanisms underlying heat-induced radiosensitization. Int J Hyperth. (2004) 20:163–74. doi: 10.1080/02656730310001619514 15195511

[98] Lee S Son B Park G Kim H Kang H Jeon J. Immunogenic effect of hyperthermia on enhancing radiotherapeutic efficacy. Int J Mol Sci. (2018) 19:2795. doi: 10.3390/ijms19092795 30227629 PMC6164993

[99] Datta NR Rogers S Ordóñez SG Puric E Bodis S. Hyperthermia and radiotherapy in the management of head and neck cancers: A systematic review and meta-analysis. Int J Hyperth. (2016) 32:31–40. doi: 10.3109/02656736.2015.1099746 26928474

[100] Valdagni R Amichetti M. Report of long-term follow-up in a randomized trial comparing radiation therapy and radiation therapy plus hyperthermia to metastatic lymph nodes in stage IV head and neck patients. Int J Radiat Oncol Biol Phys. (1994) 28:163–9. doi: 10.1016/0360-3016(94)90154-6 8270437

[101] Overgaard J Dahl O Arcangeli G. ESHO 2–85. Hyperthermia as an adjuvant to radiation therapy in the treatment of advanced neck nodes: A randomized multicenter study by the european society for hyperthermic oncology. Acta Oncol. (2024) 63:943–9. doi: 10.2340/1651-226X.2024.41035 PMC11656068 39665330

[102] Hurwitz MD. Hyperthermia and immunotherapy: clinical opportunities. Int J Hyperthermia. (2019) 36:4–9. doi: 10.1080/02656736.2019.1653499 31795827

[103] Ramsay D Stevenson H Jerjes W. From basic mechanisms to clinical research: photodynamic therapy applications in head and neck Malignancies and vascular anomalies. J Clin Med. (2021) 10:4404. doi: 10.3390/jcm10194404 34640423 PMC8509369

[104] Allison RR Moghissi K. Photodynamic therapy (PDT): PDT mechanisms. Clin Endosc. (2013) 46:24–9. doi: 10.5946/ce.2013.46.1.24 PMC3572346 23422955

[105] Biel MA. Photodynamic therapy of head and neck cancers. Methods Mol Biol Clifton NJ. (2010) 635:281–93. doi: 10.1007/978-1-60761-697-9_18 20552353

[106] Lambert A Nees L Nuyts S Clement P Meulemans J Delaere P. Photodynamic therapy as an alternative therapeutic tool in functionally inoperable oral and oropharyngeal carcinoma: A single tertiary center retrospective cohort analysis. Front Oncol. (2021) 11:626394. doi: 10.3389/fonc.2021.626394 33747943 PMC7970031

[107] Ahn PH Quon H O’Malley BW Weinstein G Chalian A Malloy K. Toxicities and early outcomes in a phase 1 trial of photodynamic therapy for premalignant and early stage head and neck tumors. Oncol. (2016) 55:37–42. doi: 10.1016/j.oraloncology.2016.01.013 PMC4943020 26865261

[108] Hosokawa S Takebayashi S Takahashi G Okamura J Mineta H. Photodynamic therapy in patients with head and neck squamous cell carcinoma. Lasers Surg Med. (2018) 50:420–6. doi: 10.1002/lsm.22802 29399863

[109] Hosokawa S Takahashi G Sugiyama K-I Takebayashi S Okamura J Takizawa Y. Porfimer sodium-mediated photodynamic therapy in patients with head and neck squamous cell carcinoma. Photodiagn Photodyn Ther. (2020) 29:101627. doi: 10.1016/j.pdpdt.2019.101627 31866532

[110] Gunaydin G Gedik ME Ayan S. Photodynamic therapy—Current limitations and novel approaches. Front Chem. (2021) 9:691697. doi: 10.3389/fchem.2021.691697 34178948 PMC8223074

[111] Stolik S Delgado JA Pérez A Anasagasti L. Measurement of the penetration depths of red and near infrared light in human “Ex vivo” Tissues. J Photochem Photobiol B. (2000) 57:90–3. doi: 10.1016/s1011-1344(00)00082-8 11154088

[112] Vaupel P Thews O Hoeckel M. Treatment resistance of solid tumors: role of hypoxia and anemia. Med Oncol Northwood Lond Engl. (2001) 18:243–59. doi: 10.1385/MO:18:4:243 11918451

[113] Xiao Z Halls S Dickey D Tulip J Moore RB. Fractionated versus standard continuous light delivery in interstitial photodynamic therapy of dunning prostate carcinomas. Clin Cancer Res. (2007) 13:7496–505. doi: 10.1158/1078-0432.CCR-07-1561 18094434

[114] National Cancer Institute (NCI). A Phase III Randomized Study of Maintenance Nivolumab Versus Observation in Patients With Locally Advanced, Intermediate Risk HPV Positive OPSCC (2024). Available online at: clinicaltrials.gov (Accessed December 23, 2024).

[115] Canadian Cancer Trials Group. Randomized Phase II Study of Cisplatin Plus Radiotherapy Versus Durvalumab Plus Radiotherapy Followed by Adjuvant Durvalumab Versus Durvalumab Plus Radiotherapy Followed by Adjuvant Tremelimumab and Durvalumab in Intermediate Risk HPV-Positive Locoregionally Advanced Oropharyngeal Squamous Cell Cancer (LA-OSCC) (2024). Available online at: clinicaltrials.gov (Accessed December 23, 2024).

[116] Nanobiotix A Phase 3 Study of NBTXR3 Activated by Investigator&x27;s Choice of Radiotherapy Alone or Radiotherapy in Combination With Cetuximab for Platinum-Based Chemotherapy-Ineligible Elderly Patients With LA-HNSCC (2024). Available online at: clinicaltrials.gov (Accessed December 23, 2024).

[117] Mehanna H Robinson M Hartley A Kong A Foran B Fulton-Lieuw T. Radiotherapy plus cisplatin or cetuximab in low-risk human papillomavirus-positive oropharyngeal cancer (De-ESCALaTE HPV): an open-label randomised controlled phase 3 trial. Lancet. (2019) 393:51–60. doi: 10.1016/S0140-6736(18)32752-1 30449623 PMC6319250

[118] Gebre-Medhin M Brun E Engström P Haugen Cange H Hammarstedt-Nordenvall L Reizenstein J. ARTSCAN III: A randomized phase III study comparing chemoradiotherapy with cisplatin versus cetuximab in patients with locoregionally advanced head and neck squamous cell cancer. J Clin Oncol. (2021) 39:38–47. doi: 10.1200/JCO.20.02072 33052757 PMC7771720

[119] Rischin D King M Kenny L Porceddu S Wratten C Macann A. Randomized trial of radiation therapy with weekly cisplatin or cetuximab in low-risk HPV-associated oropharyngeal cancer (TROG 12.01) - A trans-tasman radiation oncology group study. Int J Radiat Oncol Biol Phys. (2021) 111:876–86. doi: 10.1016/j.ijrobp.2021.04.015 34098030

[120] Gillison ML Trotti AM Harris J Eisbruch A Harari PM Adelstein DJ. Radiotherapy plus cetuximab or cisplatin in human papillomavirus-positive oropharyngeal cancer (NRG oncology RTOG 1016): A randomised, multicentre, non-inferiority trial. Lancet Lond Engl. (2019) 393:40–50. doi: 10.1016/S0140-6736(18)32779-X PMC6541928 30449625

[121] Ang KK Zhang Q Rosenthal DI Nguyen-Tan PF Sherman EJ Weber RS. Randomized Phase III Trial of Concurrent Accelerated Radiation plus Cisplatin with or without Cetuximab for Stage III to IV Head and Neck Carcinoma: RTOG 0522. J Clin Oncol. (2014) 32:2940–50. doi: 10.1200/JCO.2013.53.5633 PMC4162493 25154822

[122] Beitler JJ Zhang Q Fu KK Trotti A Spencer SA Jones CU. Final results of local-regional control and late toxicity of RTOG 9003: A randomized trial of altered fractionation radiation for locally advanced head and neck cancer. Int J Radiat Oncol Biol Phys. (2014) 89:13–20. doi: 10.1016/j.ijrobp.2013.12.027 24613816 PMC4664465

[123] Nguyen-Tan PF Zhang Q Ang KK Weber RS Rosenthal DI Soulieres D. Randomized phase III trial to test accelerated versus standard fractionation in combination with concurrent cisplatin for head and neck carcinomas in the radiation therapy oncology group 0129 trial: long-term report of efficacy and toxicity. J Clin Oncol. (2014) 32:3858–66. doi: 10.1200/JCO.2014.55.3925 PMC4239304 25366680

[124] Lyhne NM Primdahl H Kristensen CA Andersen E Johansen J Andersen LJ. The DAHANCA 6 randomized trial: effect of 6 vs 5 weekly fractions of radiotherapy in patients with glottic squamous cell carcinoma. Radiother Oncol. (2015) 117:91–8. doi: 10.1016/j.radonc.2015.07.004 26255764

[125] Overgaard J Hansen HS Specht L Overgaard M Grau C Andersen E. Five compared with six fractions per week of conventional radiotherapy of squamous-cell carcinoma of head and neck: DAHANCA 6&7 randomised controlled trial. Lancet. (2003) 362:933–40. doi: 10.1016/S0140-6736(03)14361-9 14511925

[126] Overgaard J Mohanti BK Begum N Ali R Agarwal JP Kuddu M. Five versus Six Fractions of Radiotherapy per Week for Squamous-Cell Carcinoma of the Head and Neck (IAEA-ACC Study): A Randomised, Multicentre Trial. Lancet Oncol. (2010) 11:553–60. doi: 10.1016/S1470-2045(10)70072-3 20382075

[127] Corvò R Benasso M Sanguineti G Lionetto R Bacigalupo A Margarino G. Alternating chemoradiotherapy versus partly accelerated radiotherapy in locally advanced squamous cell carcinoma of the head and neck: results from a phase III randomized trial. Cancer. (2001) 92:2856–67. doi: 10.1002/1097-0142(20011201)92:11<2856::aid-cncr10132>3.0.co;2-6 11753959

[128] Olmi P Crispino S Fallai C Torri V Rossi F Bolner A. Locoregionally Advanced Carcinoma of the Oropharynx: Conventional Radiotherapy vs. Accelerated Hyperfractionated Radiotherapy vs. Concomitant Radiotherapy and Chemotherapy–a Multicenter Randomized Trial. Int J Radiat Oncol Biol Phys. (2003) 55:78–92. doi: 10.1016/s0360-3016(02)03792-6 12504039

[129] Ghosh-Laskar S Kalyani N Gupta T Budrukkar A Murthy V Sengar M. Conventional Radiotherapy versus Concurrent Chemoradiotherapy versus Accelerated Radiotherapy in Locoregionally Advanced Carcinoma of Head and Neck: Results of a Prospective Randomized Trial. Head Neck. (2016) 38:202–7. doi: 10.1002/hed.23865 25224814

